# Repetition of the Remedy

**Published:** 1889-05

**Authors:** 


### REPETITION OF THE REMEDY.

(From Bœnninghausen's *Aphorisms*. Translated by A. McNeil.)

The foundation of all diseases rests on an internal, immaterial, purely dynamic affection of the vital power, which is either limited to one organ or the entire organism may be affected. And if heterogenous or effete matter is present in the system, with the exception of that which is introduced from without, it is to be considered as the result of that disturbance of the vital activity and not at all as the actual cause of the disease, the driving out of which is necessary to the restoration to health.

To oppose these natural diseases we employ those agents which are designated medicines, to differentiate them from the purely nourishing substances which we call foods. These medicines have purely dynamic, sick-making properties, whereby they have the power of reproducing such diseases in their external similarity as nature herself does, although they do not necessarily possess the same mysterious, intrinsic character which is and always will remain concealed from us by an impenetrable veil.

It has been proven by constant experience, although it cannot be demonstrated by logical reasoning, that medicines generally possess the property of curing certain diseases. In answering the question, Under what conditions is this done? the two schools diverge, although hitherto they have been in unison, as the allopaths accept as their guide the formula of *contraria contrarias*, and the homœopaths that of *similia similibus*. However, they again agree with each other that only by exciting the vital power by suitable medicine can recovery be obtained, and that without this vital power and its reaction, every curative agent must be completely unefficacious.

In this active reaction of the vital power, we homœopaths perceive the foundation on which rests what we call the primary and secondary action of drugs. The primary action of a medicine is that which follows when its sick-making property makes its direct attack on the living organism. The secondary action consists in the reaction of the active vital power against the assault made on it. The two kinds of action stand in direct opposition to each other, and although each one is to be considered as the result of the mutual dynamic power of the vitality and of the medicine, yet they manifest differences in their struggle against each other which an experienced eye easily recognizes.

The complete cure of a disease is, consequently, the direct result of the secondary action in which the living and constantly reacting organism obtains more and more the upper hand in its struggle against the medicine, until it (the medicine) and with it the natural disease (in whose stead it appears) is entirely subdued and annihilated, and thereby health is restored.

From this it is easily perceived how careful the homœopathic physician must be not to interfere in the contest between the primary and secondary action, either by the administration of new doses of medicine to aid that already given and thereby to inevitably prolong the strife. Therefore, in our opinion, nothing is more dangerous and pernicious for the physician than impatience; and he will never repent waiting quietly as long as he sees the conflict in full activity, which he perceives by his accurate knowledge of the peculiar or characteristic symptoms, and that there is no change of the indications calling for another medicine. In this latter case, which does not often occur, there are the most positive criteria and cautions to guide him, and he will scarcely run the risk of either an injurious haste or of a hurtful delay.

It remains to be mentioned briefly that the period of waiting after the primary action of a drug is extremely different according to the nature of the medicine and the character of the case. While in the most acute diseases, as cholera, for instance, the time is measured by minutes, and in the most painful sufferings instant relief and a rapid removal is possible, yet, in chronic diseases often entire weeks pass before the health-bringing secondary action begins to manifest itself. And it is in just these old, long-continued chronic complaints that a too hasty repetition or a too early administration of a new remedy is the most injurious, often to such a degree that the harm can scarcely be overcome, and then only after a great loss of time. It is on this rock that the beginner in Homœopathy is most likely to be wrecked; and also those who have long served under the flag of allopathy, for *quo est imbuta recens, servabit odorem testa diu!*

